# The Appeal of the Insane to Science

**Published:** 1892-05-14

**Authors:** 


### The Doctor's Armchair.

THE APPEAL OF THE INSANE TO SCIENCE.

The Edinburgh University Press has just issued the fourth part of the first volume of Dr. Byrom Bramwell's *Atlas of Clinical Medicine.* Four coloured plates are published therewith, each showing a life-like portrait of an insane person. It is impossible to imagine anything more tragically suggestive than these four pictorial representations of mad men and women. The wreck of reason has reduced them all, not to animal imbecility, but to a kind of conscious desperation. The first plate represents a woman suffering from melancholia. The face expresses hopeless, helpless despair. The second picture is also that of a woman. She stands with hands clasped, a melancholy brow, and large, piteous eyes turned upwards appealingly, as if to invoke a steely heaven which experience has taught her will not or cannot help. The third is the portrait of a man. Despair and determination have obtained a joint mastery over his features: suicide is his purpose, a purpose which, sooner or later, he will unfailingly carry out. The fourth face is, in some respects, the most tragic of all. It is that of a woman in middle life, with regular features, and a countenance that has once been comely. She is not melancholy, but a raving maniac. The defiant eyes, and the open mouth, filled with mad laughter, show a kind of riotous delight in the wreck and abandonment of reason, conscience, and womanhood. These are men and women who once were sane and sober like ourselves: we who look at them may, before life is over, be melancholy, desperate, and mad as they now are.

What is the great hope of the world among cultured peoples in these later days? What is it we are all more or less consciously and steadily looking to for deliverance from many of the preventable ills which six thousand years of historic time have gradually brought upon us? It is science we look to, and science only: some of us with little faith, some with much, and some with none. A great many of the grinders in the mill of life have recognised their duty of grinding, and have resolved to do it whilst life shall last without any other hope than the recompense of the grinders' weekly wages. They have no hope, but they retain a stolid, mechanical sanity. But the mill of life grinds many so sternly that they cannot retain their sanity—it is ground out of them. They yield themselves up to the melancholy of unresisted despair; or to the sharper misery of unanswered appeals to heaven; or to the defiance of the riotous maniac.

There is a practical problem here for science to solve. If any man could speedily cure or entirely prevent the brain degeneration which results in the loss of reason, he would be the chief priest and pontiff of all the medicine which the world has ever seen. Compared with madness, cancer and consumption are diseases which may be accepted with resignation, and almost with joy. Whilst reason still occupies her throne, philosophy and religion can endure the worst of human ills with fortitude, and even hope. But when the mind is gone all is gone. Death itself is a joyful occasion compared with the ruin that madness brings upon men.

To science in the abstract an appeal will not lie; there is no such thing as science in the abstract; science is concrete and tangible in the living scientific men of the time; it is to these that appeals must be made, made separately, and one by one. Can anything be done for the mad? Much; and much is done! But if there is any disease which science ought to rouse herself to prevent rather than to cure, madness is that disease. It is as indisputable a proposition as that snow will fall in winter in the Arctic regions, to say that madness is a preventable disease. If all the world were as capable as the most capable men that are in it, every form of insanity might be stamped out in a single century. Men are mad because they have had mad ancestors. But if young men and women with a taint of hereditary insanity were not permitted to become progenitors of their kind, hereditary madness, the gravest of all forms of insanity, would almost immediately disappear.

The too great pressure of life in our modern states is a fruitful cause of madness. Does any inconsequent person imagine that the human race has not wit enough to develop and construct a civilisation which shall free most of us from this too great pressure? It has wit, and to spare. But it has not generosity enough; and the present race of scientific men are by no means the most generous of the human species. In practical generosity they are leagues and centuries behind the unintellectual religionists whose minds they profess to despise. A more generous science could work miracles for men.

The third most common cause of madness is deficient nutrition of the brain, a form of physical starvation, due sometimes to the actual want of food, and sometimes to ignorant and foolish methods of feeding. To argue that this is an obviously preventable cause of madness would be waste of time. It is as preventable as a great conflagration is by the removal of all combustible materials from the neighbourhood.

It is, we admit, an unparalleled demand that the ills we have spoken of make upon men of science. The demand is, that these ills shall be prevented; or where they cannot be prevented, that they shall be promptly cured. But prevention is the ambition most worthy of a great, wide-seeing, and magnanimous science. Religion has had her chances; but she has only cured the ills of the world to a strictly limited extent. Science has now her opportunity! Will she fail, or will she succeed? She will fail if she fails of a generous and magnanimous temper. Self-seeking may be her ruin; pedantry may be her death-blow. Men cannot change the order of things into which they have been born. Destiny has fixed us all here, scientific men among the rest, to see what kind of stuff each one of us is made of. The unseen powers vivisect us, and peer at us through their microscopes, and subject us to their analysis and synthesis exactly as we deal with those creatures over whom we have authority and power. Grant that it is posterity alone which will pronounce judgment and sentence upon us, yet posterity can sink us to the Hades of those whose names are not worth preserving, because their work has been poor and worthless; or to the still worse Gehenna of those whose doings have been foolish and bad. What is to be done to ennoble the minds of all scientific men? It is a great problem. The common world, sane and mad, asks science for all kinds of living bread. Will science give it little but incomprehensible and uneatable stones?

